# Association of Diabetes with Heart Rate Variability during Hemodialysis: Insights from the Frequent Hemodialysis Network Daily Trial

**DOI:** 10.34067/KID.0000000765

**Published:** 2025-03-13

**Authors:** Bróna M. Moloney, Glenn M. Chertow, Finnian R. Mc Causland

**Affiliations:** 1 Brigham and Women's Hospital, Boston, Massachusetts; 2 Harvard Medical School, Boston, Massachusetts; 3 Departments of Medicine, Epidemiology and Population Health, and Health Policy, Stanford University School of Medicine, Stanford, California

**Keywords:** cardiovascular, chronic dialysis, clinical trial, daily hemodialysis, diabetes

## Abstract

**Key Points:**

- Patients with diabetes on hemodialysis had 18% lower SD of the normal-to-normal R-R interval, a proxy for lower heart rate variability.
- The association of diabetes with intradialytic hypotension was not mediated by SD of the normal-to-normal R-R interval.
- Targeted therapies to mitigate autonomic neuropathy in patients with diabetes on hemodialysis warrant further investigation.

**Background:**

Autonomic dysfunction is common among patients with diabetes receiving hemodialysis. We wished to explore the association of diabetes with heart rate variability (HRV; a surrogate of autonomic dysfunction) and whether HRV mediates the association of diabetes with intradialytic hypotension (IDH).

**Methods:**

In this secondary analysis of the Frequent Hemodialysis Network Daily Trial, we performed the following: (*1*) random effects linear regression to estimate the association of diabetes with log-transformed low-frequency (LF) power (proxy of sympathetic activity), high-frequency (HF) power (proxy of parasympathetic activity), ratio of LF/HF (proxy for sympathovagal balance), and SD of the normal-to-normal R-R interval (SDNN, measured at baseline and 12 months) and (*2*) linear regression to explore the association of diabetes with changes in HRV parameters over 12 months. Models were adjusted for age, sex, designated race, height, access type, hemodialysis vintage, history of heart failure, prehemodialysis systolic BP, heart rate, ultrafiltration rate, hemoglobin, serum albumin, *β*-blocker use, calcium channel blocker use, diuretic use, left ventricular mass, and randomized treatment assignment.

**Results:**

Of the 198 patients without baseline atrial fibrillation, 82 (41%) had self-reported diabetes. In adjusted random effects models, diabetes (versus no diabetes) was associated with lower SDNN −18% (95% confidence interval [CI], −27 to −9) on a per session basis. The presence of diabetes was not associated with differences in LF 7% (95% CI, −20 to 43), HF 10% (95% CI, −10 to 33), or LF/HF −4% (95% CI, −19 to 14). Diabetes (versus no diabetes) was not associated with a change from baseline to 12 months in any HRV parameter. SDNN did not attenuate the observed association of diabetes with IDH.

**Conclusions:**

Among participants in the Frequent Hemodialysis Network Daily Trial, diabetes (versus no diabetes) was associated with 18% lower SDNN. The association of diabetes with IDH did not seem to be mediated by SDNN. The reasons for higher rates of IDH in patients with diabetes remain elusive.

**Clinical Trial registry name and registration number:**

NCT00264758.

## Visual Abstract

**Figure:**
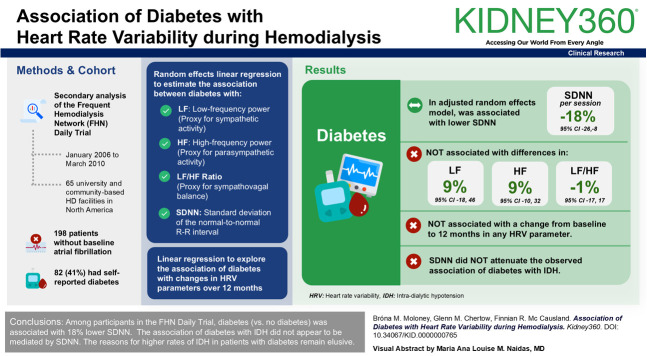

## Introduction

Autonomic dysfunction is a common complication among patients with long-standing diabetes^1^ but is also well-described among patients with kidney failure, independent of diabetes status.^2–4^ Autonomic dysfunction generally reflects greater disease severity and is associated with a higher risk of adverse cardiovascular outcomes and death among patients receiving maintenance hemodialysis. As such, the combined presence of diabetes and kidney failure may predispose to a higher frequency and severity of autonomic dysfunction and its sequelae.

Intradialytic hypotension (IDH) complicates approximately 5%–40%^5^ of hemodialysis sessions, depending on the definition used, and is associated with mortality.^6,7^ Of note, IDH is associated with maladaptive changes in cardiac structure because of reduced myocardial perfusion and stunning,^8^ thereby rendering the myocardium a vulnerable substrate, prone to cardiac arrhythmia.^9^ We previously observed that self-reported diabetes (versus no diabetes) was associated with a higher rate of IDH (incidence rate ratio, 1.93; 95% confidence interval [CI], 1.26 to 2.95), among participants in the Frequent Hemodialysis Network (FHN) Daily Trial.^10^ Although the overall etiology of IDH is likely multifactorial, we and others have postulated that autonomic dysfunction may contribute to its risk.

Abnormal responses in heart rate variability (HRV) are widely accepted surrogate biomarkers for autonomic dysfunction and have been associated with adverse changes in cardiac structure and cardiovascular mortality among patients receiving maintenance hemodialysis.^11–17^ Therefore, in a secondary analysis of the FHN Daily Trial, we explored if patients with diabetes would have lower HRV compared with patients without diabetes and if the association of diabetes with IDH was mediated by HRV.

## Methods

### Study Population and Design

The FHN Trial (NCT00264758; registered December 12, 2005), conducted from January 2006 to March 2010, was a multicenter, randomized, parallel-group study conducted at 65 university and community-based hemodialysis facilities in North America. The study design, protocol, and primary results have been published.^18^ The trial aimed to compare the efficacy of frequent hemodialysis (six times per week) versus conventional hemodialysis (three times per week) administered in-center.^18^ The protocol for the FHN Daily Trial received approval from the Institutional Review Boards at all participating centers. The study was conducted in accordance with the principles of the Declaration of Helsinki. Before enrollment, all individuals provided written informed consent in accordance with ethical guidelines. Deidentified data used for this analysis were sourced from the National Institute of Diabetes and Digestive and Kidney Diseases (NIDDK) FHN study data repository.

Patients eligible for inclusion in the study had kidney failure requiring maintenance hemodialysis, were older than 13 years, weighed more than 30 kg, and demonstrated a mean equilibrated Kt/V >1.0 for two baseline hemodialysis sessions preceding enrollment. Notable exclusion criteria included residual urea clearance >3 ml/min per 35 L, medical necessity for hemodialysis >3 times per week, a requirement for daily or nocturnal hemodialysis within the previous 3 months or allograft failure necessitating hemodialysis within the past 3 months, and life expectancy of less than 6 months. For this study, we excluded patients with a history of atrial fibrillation (*n*=11) or who did not have HRV measurements available (*n*=38), leaving a total of *n*=198. A comparison of baseline characteristics between included and excluded patients is presented in Supplemental Table 1.

The trial evaluated two composite coprimary outcomes: (*1*) death or 12-month change in left ventricular mass, as determined by cardiac MRI, and (*2*) death or 12-month change in the Physical Health Composite score from the RAND 36-Item Health Survey.

### Exposure Variable for Present Analyses

The exposure of interest for the present analyses was the presence or absence of a baseline diagnosis of diabetes, as reported by the site investigators.

### Outcome Variables for the Present Analyses—HRV Measurements

HRV measurements were abstracted from 24-hour Holter monitor recordings performed at baseline and 12-month visits. Where possible, the 24-hour Holter recording was obtained on the first hemodialysis day after a weekend and the device was fit to study participants at any point in the hour preceding the start of hemodialysis.

The primary outcome measure for this analysis was the SD of the normal-to-normal (N-N) R-R intervals (SDNN). These intervals represent the time elapsed between consecutive normal heartbeats, measured from the peak (R-wave) of one QRS complex to the peak of the next QRS complex. The use of N-N interval (*as opposed to R-R interval*) excludes any premature beats or irregular heart rhythms and gives an estimate of overall HRV. SDNN is a surrogate for both sympathetic and parasympathetic activity, measured in milliseconds (ms), where higher SDNN values are a proxy for greater HRV (in turn, a proxy for better autonomic function).^19^ Secondary outcomes measures included: (*1*) low-frequency (LF) power spectrum value, a biomarker of sympathetic activity^20,21^; (*2*) high-frequency (HF) power spectrum value, a biomarker of parasympathetic activity^19^; and (*3*) LF/HF ratio, a marker of sympathovagal balance.^19,22^ For reference, reported “normal” mean values of these parameters are 141±39 ms, 1170±416 ms^2^, 975±203 ms^2^, and 1.5–2.0, for SDNN, LF, HF, and LF/HF ratio, respectively.^19^ We also measured changes in SDNN and LF, HF, and LF/HF ratio from baseline to 12 months as additional outcomes of interest.

### Statistical Analysis

We reported continuous variables as means (±SD) for normally distributed data or as medians (25th–75th percentiles) for non-normally distributed data. We reported categorical variables using frequency distribution and proportions. We compared baseline characteristics according to diabetes status using the Student *t* test, Mann–Whitney *U* test, or Pearson chi-squared test, as appropriate for data distribution. Owing to positive skewness, we log-transformed SDNN, LF, HF, and their change from baseline to 12 months for analyses.

We fit unadjusted and adjusted random effect linear regression models (model assumes a random intercept for each panel and homoskedasticity with no serial correlation within each panel) to explore the association of diabetes (versus none) with metrics of HRV. In our multivariable model, we adjusted for age, sex, designated race (Black versus non-Black), height, vascular access, duration of kidney failure, history of heart failure, predialysis systolic BP, heart rate, ultrafiltration rate, hemoglobin, serum albumin, *β*-blocker use, calcium channel blocker use, diuretic use, baseline left ventricular mass, and randomized treatment assignment. Analogous linear regression models were fit to explore the association of diabetes status with change in HRV parameters from baseline to 12 months, with additional adjustment for the baseline measurement of the corresponding HRV parameter.

For mediation analyses (random effects regression to evaluate associations on a per-session basis) exploring if SDNN mediates the association of diabetes with IDH, the following conditions had to be satisfied, according to the framework of Barron and Kenny^23^: (*1*) there must be a significant association of the exposure (diabetes) with the outcome (decline from prehemodialysis to nadir intrahemodialysis systolic BP); (*2*) there must be a significant association of the exposure (diabetes) with the potential mediator (SDNN); and (*3*) there must be an attenuation of the association of the exposure (diabetes) with the outcome after adjusting for the potential mediator. Ultimately, these conditions were not met; as such, a formal mediation analysis could not be performed (Supplemental Figure 1 and Supplemental Table 2).

We considered two-tailed *P* values < 0.05 as statistically significant, without adjustment for multiple testing. Missing data were not imputed. We conducted all analyses using Stata MP (version 18.0, Stata Corp., College Station, TX).

## Results

### Baseline Characteristics

Of the 198 FHN Daily Trial participants included in the present analyses, the mean age was 55±12 years, and 36 (44%) were women. Eighty-two (41%) had diabetes at baseline. Patients with diabetes were more likely to be older, have higher prehemodialysis systolic BP, higher posthemodialysis weight, and be prescribed diuretics, compared with patients without diabetes. They were also more likely to have a shorter hemodialysis vintage and to have lower hemoglobin and serum albumin (Table 1).

**Table 1:** Baseline characteristics according to diabetes status

Characteristics	No Diabetes (*n*=116)	Diabetes (*n*=82)	*P*-value
Age, yr	46±13	55±12	*P* < 0.001
Female, *n* (%)	42 (36.2)	36 (43.9)	*P* = 0.28
Black, *n* (%)	61 (52.6)	29 (35.4)	*P* = 0.02
Height, cm	168±10	168±11	*P* = 0.73
Posthemodialysis weight, kg	74±19	84±21	*P* < 0.001
**Vascular access, *n* (%)**			*P* = 0.04
Graft	18 (15.9)	15 (19.0)	
Fistula	79 (69.9)	42 (53.2)	
Catheter	16 (14.2)	22 (27.8)	
**Dialysis vintage, yr, *n* (%)**			*P* = 0.01
0 to <2	23 (19.8)	33 (40.2)	
2–5	38 (32.8)	23 (28.0)	
>5	55 (47.4)	26 (31.7)	
History of heart failure, *n* (%)	17 (14.7)	20 (24.4)	*P* = 0.08
Prehemodialysis systolic BP, mm Hg	144±17	150±19	*P* = 0.01
Heart rate, bpm	75±15	76±12	*P* = 0.55
Ultrafiltration rate, ml/kg per hour	12.0±3.8	11.1±4.2	*P* = 0.09
Hemoglobin, g/dl	12.1±1.2	11.7±1.2	*P* = 0.01
Albumin, g/dl	4.1±0.4	3.8±0.4	*P* < 0.001
CCB use, *n* (%)	56 (48.3)	49 (59.8)	*P* = 0.11
*β*-blocker use, *n* (%)	72 (62.1)	47 (57.3)	*P* = 0.50
Diuretic use, *n* (%)	9 (7.8)	15 (18.3)	*P* = 0.03
LV mass, g/m^2^	145±62	139±46	*P* = 0.45
Randomized to 6/wk hemodialysis, *n* (%)	61 (52.6)	42 (51.2)	*P* = 0.85

Results are presented as mean±SD, or median (25th–75th percentiles) for continuous variables. CCB, calcium channel blocker; LV, left ventricle.

### Association of Diabetes with the SDNN

**Figure 1:**
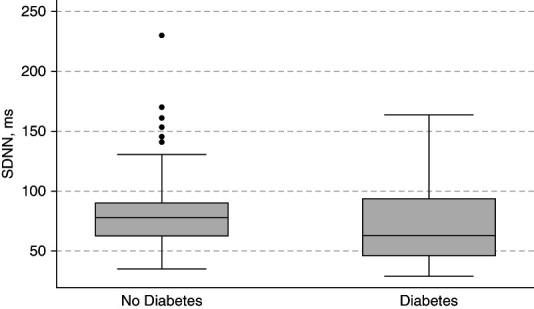
**Distribution of SDNN according to baseline diabetes.** Distribution of average SDNN according to baseline diabetes status. Upper, middle, and lower lines of the box represent the 75th, 50th, and 25th percentiles of the distribution. SDNN, SD of the normal-to-normal R-R interval.

The median SDNN was 62 ms (43–99) among patients with diabetes and 76 ms (59–92) among patients without diabetes (Figure 1). In unadjusted, random effects models, the presence of diabetes was associated with a 15% lower SDNN (−15%; 95% CI, −23% to −6%) as compared with the absence of diabetes. In the fully adjusted model, the difference in SDNN was accentuated (−18%; 95% CI, −27% to −9%; Table 2). There was no suggestion of differential associations according to randomized treatment assignment (*P* interaction = 0.23; Supplemental Table 3).

**Table 2:** Association of diabetes with heart rate variability parameters

Outcome	Percent Difference in Outcome for DM versus Non-DM (95% CI)	*P* Value
**SDNN, ms**		
Unadjusted	−15 (−23 to −6)	<0.001
Adjusted	−18 (−27 to −9)	<0.001
**LF component, ms** ^**2**^		
Unadjusted	−5 (−26 to 21)	0.66
Adjusted	7 (−20 to 43)	0.67
**HF component, ms** ^**2**^		
Unadjusted	1 (−15 to 20)	0.92
Adjusted	10 (−10 to 33)	0.36
**LF/HF ratio**		
Unadjusted	−7 (−19 to 8)	0.33
Adjusted	−4 (−19 to 14)	0.63

The multivariable repeated measures model (using Holter data from baseline and 12 months) adjusted for age, sex, race (Black versus non-Black), height, access type, vintage (<2, 2–5, and >5 years), heart failure, predialysis systolic BP, heart rate, ultrafiltration rate, hemoglobin, albumin, *β*-blocker, calcium channel blocker, diuretics, left ventricle mass, and randomized treatment assignment. All outcomes were log-transformed for use in models estimating the difference according to the presence or absence of diabetes at baseline; the results are expressed as percentage changes in geometric means. CI, confidence interval; DM, diabetes mellitus; HF, high-frequency; LF, low-frequency; SDNN, SD of the normal-to-normal R-R interval.

### Association of Diabetes with LF, HF, and LF/HF Ratio

The median LF spectrum value was 104 (49–200) ms^2^ among patients with diabetes and 118 (46–207) ms^2^ among patients without diabetes. In unadjusted analysis, the presence of diabetes was associated with a nonsignificant 5% lower LF spectrum value (−5%; 95% CI, −26% to +21%) compared with the absence of diabetes. In adjusted analyses, the effect estimates remained nonsignificant (+7%; 95% CI, −20% to +43%; Table 2). Although there was a suggestion of differential associations according to randomized treatment assignment (*P* interaction = 0.01), subgroup estimates for 6/wk and 3/wk hemodialysis were both nonsignificant (Supplemental Table 3).

The median HF spectrum value was 38 (29–55) ms^2^ among patients with diabetes and 44 (30–59) ms^2^ among patients without diabetes. In unadjusted analysis, using random effects models, diabetes (versus no diabetes) was unassociated with HF spectrum value (point estimate, +1%; 95% CI, −15% to +20%). In adjusted analysis, the effect estimates remained nonsignificant (+10%; 95% CI, −10% to +33%; Table 2). There was no suggestion of differential associations according to randomized treatment assignment (*P* interaction = 0.19; Supplemental Table 3).

The median LF/HF ratio was 2.2 (95% CI, 1.3 to 3.6) among patients with diabetes and 2.5 (95% CI, 1.5 to 3.9) among patients without diabetes. In unadjusted analysis, diabetes was associated with a nonsignificant 7% lower LF/HF ratio (−7%; 95% CI, −19% to +8%), compared with patients without diabetes. In adjusted analyses, the small numerical differences were attenuated (point estimate, −4%; 95% CI, −19% to +14%; Table 2). There was a suggestion of differential associations according to randomized treatment assignment (*P* interaction = 0.03); subgroup estimates for 6/wk and 3/wk hemodialysis were both nonsignificant (Supplemental Table 3).

### Association of Diabetes with Change in HRV Variables from Baseline to Month 12

Baseline and 12-month visit Holter data were available for 125 patients, allowing the change in HRV variables to be examined over this period. For all parameters, there was no association between diabetes (versus no diabetes) and change in HRV parameters over 12 months in either unadjusted or adjusted analyses (Table 3).

**Table 3:** Association of diabetes with change in heart rate variability parameters over 12 months

Outcome	No Diabetes	Diabetes	Percent Difference in Outcome for DM versus Non-DM (95% CI)	
	Median (25–75th Percentile)	Median (25–75th Percentile)	Unadjusted	Adjusted
**SDNN, ms**				
Baseline	76 (58–98)	56 (44–89)	−16 (−25 to −5)	−18 (−29 to −7)
Month 12	74 (61–90)	67 (42–102)	−14 (−25 to −2)	−14 (−27 to 0)
Delta (month 12−baseline)	0 (−22 to 20)	−1 (−18 to 14)	−8 (−20 to 6)	−11 (−24 to 4)
**LF component, ms** ^**2**^				
Baseline	105 (48–194)	104 (52–210)	1 (−25 to 34)	11 (−22 to 57)
Month 12	125 (45–226)	105 (44–194)	−17 (−42 to 19)	6 (−32 to 66)
Delta (month 12−baseline)	4 (−52 to 74)	6 (−49 to 56)	−20 (−44 to 14)	−18 (−46 to 25)
**HF component, ms** ^**2**^				
Baseline	45 (29–60)	38 (30–55)	5 (−15 to 31)	13 (−12 to 46)
Month 12	43 (31–54)	38 (27–56)	−7 (−28 to 19)	10 (−18 to 47)
Delta (month 12−baseline)	−1 (−19 to 13)	−1 (−14 to 13)	−12 (−32 to 14)	−0 (−26 to 34)
**LF/HF ratio**				
Baseline	2.4 (1.5–3.9)	2.2 (1.3–4.0)	−5 (−20 to 14)	−22 (−21 to 21)
Month 12	2.8 (1.4–4.4)	2.3 (1.3–3.5)	−11 (−28 to 11)	−3 (−26 to 26)
Delta (month 12−baseline)	0.2 (−0.9 to 1.6)	0.0 (−1.4 to 1.3)	−11 (−29 to 11)	−15 (−35 to 12)

CI, confidence interval; DM, diabetes mellitus; HF, high-frequency; LF, low-frequency; SDNN, SD of the normal-to-normal R-R interval.

### Mediation Analyses

Ultimately, the *a priori* assumptions necessary to proceed with a formal (and valid) mediation analysis were not satisfied (Supplemental Figure 1 and Supplemental Table 2).

## Discussion

In this secondary analysis of the FHN Daily trial, we observed that patients with diabetes receiving maintenance hemodialysis had an 18% lower SDNN (a proxy for lower HRV and impaired autonomic function), compared with patients without diabetes, irrespective of their randomized treatment assignment (6/wk versus 3/wk). Diabetes status was not associated with a change in SDNN or other HRV parameters between baseline and 12 months. Lower SDNN did not meet criteria to be considered as a potential mediator of the association of diabetes with IDH.

The prevalence of diabetes is rising rapidly worldwide. Projected global prevalence estimates indicate that approximately 783 million persons may be living with diabetes by the year 2045.^24^ Among its many complications, diabetes predisposes to the development of autonomic neuropathy, which is particularly relevant among patients with kidney failure, where it may predispose to IDH, falls, and adverse cardiovascular events.^11,25^ Despite these known associations, there are limited data regarding differences in autonomic dysfunction measurements according to diabetes status among patients receiving dialysis. Detailed methods of measuring autonomic dysfunction, such as tilt-table testing, microneurography, or positron emission tomography with either [123-I] meta-iodobenzylguanidine washout or [^11^C]-meta-hydroxy-ephedrine uptake studies,^26–28^ are cumbersome in patients with kidney failure, many of whom are extremely frail. Thus, metrics of HRV have increasingly been used as more accessible biomarkers of sympathetic and parasympathetic activity. Indeed, lower HRV (a proxy for autonomic dysfunction) has been associated with higher cardiovascular risk and is an independent predictor of cardiac death in patients receiving maintenance hemodialysis.^29–31^

In normal individuals, there is beat-to-beat variability in heart rate, reflecting the opposing actions of the sympathetic and parasympathetic nervous systems on cardiac conduction. A lower SDNN, calculated as the SD of the R-R intervals of normal beats, is believed to be reflective of overall HRV and represents the oscillations in intervals between successive heartbeats that are modulated by both sympathetic and parasympathetic activity (although, as an overall metric, SDNN does not distinguish between the relative contributions from parasympathetic and sympathetic systems).^19^ Lower SDNN has been associated with adverse outcomes in patients with diabetes. In a secondary analysis of the Action to Control Cardiovascular Disease in Diabetes Patients study, cardiac autonomic neuropathy (defined using three different HRV composite definitions, all of which incorporated SDNN) was associated with incident heart failure.^32^ Similarly, in a prior report of patients with kidney failure referred for kidney transplantation (*N*=278), of whom 168 were on hemodialysis and 94 had diabetes, patients with diabetes had markedly lower SDNN values than their non-diabetic counterparts.^33^ Our present observations that diabetes (versus no diabetes) was associated with lower SDNN confirm and extend these observations.

We did not find an association of diabetes (versus no diabetes) with LF (reflective of sympathetic predominance), HF (reflective of parasympathetic activity), or the LF/HF ratio (a proposed measure of sympathovagal balance). Our findings corroborate, to some degree, those of a small prospective study by Giordano *et al.*, which found no differences in intradialytic HRV parameters among patients with diabetes (versus no diabetes).^12^ However, in the Giordano *et al.* study, biomarkers of HRV reflective of enhanced sympathetic activity were more pronounced in the posthemodialysis periods for patients with (versus without) diabetes. It is possible that our lack of findings for differences in LF, HF, or LF/HF ratio according to diabetes may relate to the averaging of these parameters over the entire perihemodialysis period, which may have obscured signals for differences in the prehemodialysis, intrahemodialysis, or posthemodialysis periods.

We found no association between diabetes at baseline and change in any of the HRV parameters over 12 months. At first pass, this may seem unexpected, as one might expect neuropathy and autonomic dysfunction to progress over time. Potential explanations for the lack of observed associations may relate to 12 months being too short an interval to detect changes or perhaps to the influence of other factors that may have altered the associations. In this respect, it has been hypothesized that enhanced solute clearance could mitigate the effects of uremia on autonomic dysfunction, with previous small observational studies noting improvements in nerve conduction velocity among patients receiving hemodialysis with higher clearance metrics or following kidney transplantation.^34,35^ In our analyses, the suggestion of differential associations of diabetes mellitus and hemodialysis frequency with HRV outcomes is consistent with the findings from Chan *et al.*, who observed that the benefits of 6/wk hemodialysis versus 3/wk hemodialysis on HRV parameters seemed to be restricted to patients without diabetes.^17^ Data from a small prospective cohort study that followed patients from CKD stage 4 through hemodialysis initiation similarly reported improvement in HRV parameters after hemodialysis therapy, again with more pronounced improvements among patients without diabetes.^36^ Taken together, the data suggest that abnormalities in HRV may be ameliorated by more effective (or efficient) correction of uremia with hemodialysis but that changes in HRV in patients with diabetes are immutable (or at least immutable by dialysis), perhaps related to neuropathic changes induced by longstanding diabetes before the development of kidney failure. In other words, and perhaps unsurprisingly, dialysis can correct uremic autonomic neuropathy but cannot correct diabetic autonomic neuropathy.

In our prior analyses, we observed that diabetes was independently associated with a higher risk of developing IDH among participants of the FHN Daily trial.^10^ In the present analysis, SDNN did not meet criteria to be considered as a potential mediator of this association, suggesting that alternative mechanisms are likely to be responsible, with the potential for multiple contributing etiologies in any given patient.^37^

Strengths of our study include the analysis of robustly collected data in the setting of a randomized controlled trial. This, together with repeated measures of HRV at baseline and 12 months, allowed us to perform multivariable models, adjusting for several potential confounders. However, we acknowledge that our study has several limitations. We did not have access to the raw Holter data, precluding our ability to determine the exact analysis time for each recording and to distinguish between the prehemodialysis, intrahemodialysis, or posthemodialysis periods. We acknowledge that the use of HRV parameters, particularly the frequency bands, and their interpretation in the context of their relative contributions to components of the autonomic system remain under debate. We therefore urge caution in drawing firm conclusions or inferences from the analysis of the frequency domain findings. Misclassification of diabetes is a potential issue, as some patients with diabetes who have reached ESKD no longer experience hyperglycemia or require glucose-lowering medications. Although we had extensive data on trial participants and adjusted for multiple covariates, we had no data on physical activity during the actual Holter recordings, data on baseline autonomic dysfunction, or the use of medications that might alter sympathetic or parasympathetic activity around the time of Holter monitoring; thus, residual confounding is likely. Finally, the generalizability of these findings may be limited to patients with similar characteristics to those enrolled in the FHN Daily Trial who underwent Holter monitoring. Even when accounting for observed characteristics, patients willing to participate in a randomized clinical trial where participation could dramatically increase the burden of an already burdensome therapy (6/wk versus 3/wk hemodialysis) are likely to differ from most patients with kidney failure receiving maintenance dialysis.

In summary, in this secondary analysis of the FHN Daily Trial, we observed that patients with diabetes have lower SDNN, a proxy for lower overall HRV, compared with patients without diabetes. As lower HRV is a known risk factor for cardiac arrhythmia and sudden death, our findings have clinical implications for risk stratification and highlight the need to investigate novel approaches to mitigate autonomic neuropathy among the increasing population of patients with diabetes and kidney failure.

## Supplementary Material

## Data Availability

Original data created for the study are or will be available in a persistent repository upon publication. Aggregated Data. NIDDK Repository. Data from the FHN Daily Trial ([V4]/https://doi.org/10.58020/bx72-p494) reported here are available for request at the NIDDK Central Repository Website, Resources for Research.

## Supplemental Material

Supplemental Table 1. Baseline characteristics of included versus excluded patients.

Supplemental Table 2. Assessment of assumptions needed to perform a mediation analysis.

Supplemental Table 3. Differential associations of diabetes with change in HRV parameters over 12 months according to randomized treatment assignment.

Supplemental Figure 1. Directed acyclic graph of the mediation model.
