# Supplementary material for: Association of Diabetes with Heart Rate Variability during Hemodialysis: Insights from the Frequent Hemodialysis Network Daily Trial
Source: Kidney360. 2025 Mar 13;6(7):1127–34. doi: 10.34067/KID.0000000765 (PMC12338355; doi:10.34067/KID.0000000765)
Supplement: Supplementary file 1 [file kidney360-6-1127-s001.pdf]

ASN American Society of Nephrology | JASN Journal of the American Society of Nephrology | KIDNEY360 Accessing Our World From Every Angle | CJASN Clinical Journal of the American Society of Nephrology

ASN Journal Disclosure Form

As per ASN journal policy, I have disclosed any financial relationships or commitments I have held in the past 36 months as included below. I have listed my Current Employer below to indicate there is a relationship requiring disclosure. If no relationship exists, my Current Employer is not listed.

G. Chertow reports the following:
Employer: Stanford University School of Medicine; Consultancy: Akebia, Alebund, Ardelyx, AstraZeneca, CalciMedica, Miromatrix, Panoramic, Sanifit, Toku, Unicycive, Vertex; Ownership Interest: Ardelyx, CloudCath, Durect, Eliaz Therapeutics, Outset, Renibus, Unicycive; Research Funding: NIDDK, NIAID, CSL Behring; Advisory or Leadership Role: Board of Directors, Satellite Healthcare, Co-Editor, Brenner & Rector's The Kidney (Elsevier); and Other Interests or Relationships: DSMB service: NIDDK, George Institute, Aethlon, Bayer, Mineralys, ReCor.

I understand that the information above will be published within the journal article, if accepted, and that failure to comply and/or to accurately and completely report the potential financial conflicts of interest could lead to the following: 1) Prior to publication, article rejection, or 2) Post-publication, sanctions ranging from, but not limited to, issuing a correction, reporting the inaccurate information to the authors' institution, banning authors from submitting work to ASN journals for varying lengths of time, and/or retraction of the published work.

Name: Glenn M. Chertow
Manuscript ID: K360-2024-001084R1
Manuscript Title: Association of Diabetes with Heart Rate Variability during Hemodialysis: Insights from the Frequent Hemodialysis Network Daily Trial
Date of Completion: January 22, 2025
Disclosure Updated Date: January 22, 2025

ASN American Society of Nephrology | JASN Journal of the American Society of Nephrology | KIDNEY360 Accessing Our World From Every Angle | CJASN Clinical Journal of the American Society of Nephrology

ASN Journal Disclosure Form

As per ASN journal policy, I have disclosed any financial relationships or commitments I have held in the past 36 months as included below. I have listed my Current Employer below to indicate there is a relationship requiring disclosure. If no relationship exists, my Current Employer is not listed.

F. McCausland reports the following:
Employer: Brigham and Women's Hospital; Consultancy: GlaxoSmithKline; Zydus Therapeutics Inc.; Research Funding: Research Funding paid to institution from NIDDK, Satellite Healthcare, Novartis, Lexicon, and Fifth Eye.; Honoraria: Travel Support from Bayer, Astra Zeneca; Speakers Bureau: Bayer; and Other Interests or Relationships: Expert witness fees from Rubin-Anders scientific.

I understand that the information above will be published within the journal article, if accepted, and that failure to comply and/or to accurately and completely report the potential financial conflicts of interest could lead to the following: 1) Prior to publication, article rejection, or 2) Post-publication, sanctions ranging from, but not limited to, issuing a correction, reporting the inaccurate information to the authors' institution, banning authors from submitting work to ASN journals for varying lengths of time, and/or retraction of the published work.

Name: Finnian R. McCausland
Manuscript ID: K360-2024-001084R1
Manuscript Title: Association of Diabetes with Heart Rate Variability during Hemodialysis: Insights from the Frequent Hemodialysis Network Daily Trial,
Date of Completion: January 21, 2025
Disclosure Updated Date: January 21, 2025

ASN Journal Disclosure Form

As per ASN journal policy, I have disclosed any financial relationships or commitments I have held in the past 36 months as included below. I have listed my Current Employer below to indicate there is a relationship requiring disclosure. If no relationship exists, my Current Employer is not listed.

B. Moloney reports the following:
Employer: Mass General Brigham

I understand that the information above will be published within the journal article, if accepted, and that failure to comply and/or to accurately and completely report the potential financial conflicts of interest could lead to the following: 1) Prior to publication, article rejection, or 2) Post-publication, sanctions ranging from, but not limited to, issuing a correction, reporting the inaccurate information to the authors' institution, banning authors from submitting work to ASN journals for varying lengths of time, and/or retraction of the published work.

Name: Brona Moloney
Manuscript ID: K360-2024-001084R1
Manuscript Title: Association of Diabetes with Heart Rate Variability during Hemodialysis: Insights from the Frequent Hemodialysis Network Daily Trial
Date of Completion: February 28, 2025
Disclosure Updated Date: February 28, 2025
