# Supplementary material for: Association of Diabetes with Heart Rate Variability during Hemodialysis: Insights from the Frequent Hemodialysis Network Daily Trial
Source: Kidney360. 2025 Mar 13;6(7):1127–34. doi: 10.34067/KID.0000000765 (PMC12338355; doi:10.34067/KID.0000000765)
Supplement: Supplementary file 2 [file kidney360-6-1127-s002.pdf]

SUPPLEMENTAL MATERIAL

Supplemental Table 1. Baseline characteristics of included versus excluded patients

Supplemental Table 2. Assessment of assumptions needed to perform a mediation analysis.

Supplemental Table 3. Differential associations of diabetes with change in heart rate variability parameters over 12 months according to randomized treatment assignment.

Supplemental Figure 1. Directed acyclic graph of the mediation model

Supplemental Table 1. Baseline characteristics of included versus excluded patients

	Included n=198	Excluded n=47	
**Age, years**	50 ± 13	53 ± 16	p=0.09
**Female, n(%)**	78 (39.4%)	16 (34.0%)	p=0.50
**Black, n(%)**	90 (45.5%)	12 (25.5%)	p=0.01
**Height, cm**	168 ± 10	168 ± 12	p=0.91
**Post-HD weight, kg**	78 ± 20	78 ± 23	p=0.92
**Vascular access, n(%)**			p=0.33
**Graft**	172 (87.8%)	42 (89.4%)	
**Fistula**	16 (8.2 %)	5 (10.6%)	
**Catheter**	8 (4.1 %)	0 (0.0 %)	
**Dialysis vintage, n(%)**			p=0.24
**0 to <2 years**	56 (28.3%)	9 (19.1%)	
**2 to 5 years**	61 (30.8%)	20 (42.6%)	
**>5 years**	81 (40.9%)	18 (38.3%)	
**History of heart failure, n(%)**	37 (18.7%)	12 (25.5%)	p=0.29
**Pre-HD systolic BP, mmHg**	147 ± 18	147 ± 18	p=0.99
**Heart rate, bpm**	76 ± 14	76 ± 14	p=0.98
**Ultrafiltration rate, ml/kg/hour**	11.6 ± 4.0	12.0 ± 4.7	p=0.61
**Hemoglobin, g/dL**	12.0 ± 1.2	11.9 ± 1.4	p=0.69
**Albumin, g/dL**	4.0 ± 0.4	3.8 ± 0.4	p=0.06
**CCB use, n(%)**	105 (53.0%)	19 (40.4%)	p=0.12
**Beta-blocker use, n(%)**	119 (60.1%)	26 (55.3%)	p=0.55
**Diuretic use, n(%)**	24 (12.1%)	7 (14.9%)	p=0.61
**LV mass, g/m2**	142 ± 56	135 ± 47	p=0.45
**Randomized to 6/week HD, n(%)**	103 (52.0%)	22 (46.8%)	p=0.52

Results are presented as mean ± standard deviation, or median [25th-75th percentiles] for continuous variables.
Abbreviations: HD, hemodialysis; BP, blood pressure; CCB, calcium channel blocker; LV, left ventricle.

Supplemental Table 2. Assessment of assumptions needed to perform a mediation analysis.

**Assumption**	**Approach**	**Results**	**Caveats**	**Proceed to next assumption?**
a) Variations in the exposure significantly affect variations in the presumed mediator	Multivariable adjusted model to derive adjusted effect estimate of association of DM with log-SDNN	DM (vs. no DM) associated with -0.2 (-0.3, -0.1) difference in log-SDNN	Potential for residual confounding exists	OK to proceed
b) Variations in the exposure significantly affect variations in the outcome	Multivariable adjusted model to derive adjusted effect estimate of association of DM with SBP decline	DM (vs. no DM) associated with 5.3 (2.0, 8.7) mmHg greater SBP decline	Potential for residual confounding exists	OK to proceed
c) When paths a and b are controlled, a previously significant association of the exposure and outcome is no longer significant	Multivariable adjusted model to derive adjusted effect estimate of association of DM with SBP drop, with log-SDNN as a covariate	DM (vs. no DM) was associated with 6.3 (2.7, 9.9) mmHg greater SBP decline.<br>Also, log-SDNN was non-significantly associated with 1.5 (-2.1, 5.0) mmHg SBP decline.	The effect estimate for DM (vs. no DM) was accentuated, rather than attenuated. Also, the effect estimate for log-SDNN was non-significant.	Assumptions violated; not OK to proceed.

Supplemental Table 3. Differential associations of diabetes with change in heart rate variability parameters over 12 months according to randomized treatment assignment.

	Percent difference in outcome for DM vs. non-DM (95%CI)	P-interaction by randomized arm
**SDNN, ms**		
**Overall**	-11 (-25, 4)	0.28
**3/week HD**	-15 (-36, 13)	
**6/week HD**	-14 (-34, 11)	
**Low Frequency component, ms^2**		
**Overall**	-18 (-46, 24)	0.01
**3/week HD**	28 (-33, 144)	
**6/week HD**	-45 (-72, 9)	
**High Frequency component, ms^2**		
**Overall**	1 (-25, 36)	0.15
**3/week HD**	5 (-34, 67)	
**6/week HD**	-1 (-40, 63)	
**LF/HF ratio**		
**Overall**	-16 (-35, 10)	0.03
**3/week HD**	26 (-18, 94)	
**6/week HD**	-38 (-60, -4)	

Supplemental Figure 1. Directed acyclic graph of the mediation model

Shown is a directed acyclic graph illustrating the assumed pathways relating diabetes, SDNN, and the decline in systolic blood pressure. The solid line from the exposure to the outcome represents a natural direct effect that is independent of changes in SDNN. There is also an indirect effect that is potentially mediated through changes in log-transformed SDNN. Comparing the relative magnitude of the direct and indirect effects allows an estimation of the role that changes in SDNN play with respect to changes in systolic blood pressure. Covariates that were considered in models included age, sex, risk, height, vascular access, dialysis vintage, heart failure, pre-dialysis systolic blood pressure, heart rate, ultrafiltration rate, hemoglobin, albumin, calcium channel blocker use, diuretic use, left ventricular mass, randomized treatment assignment.

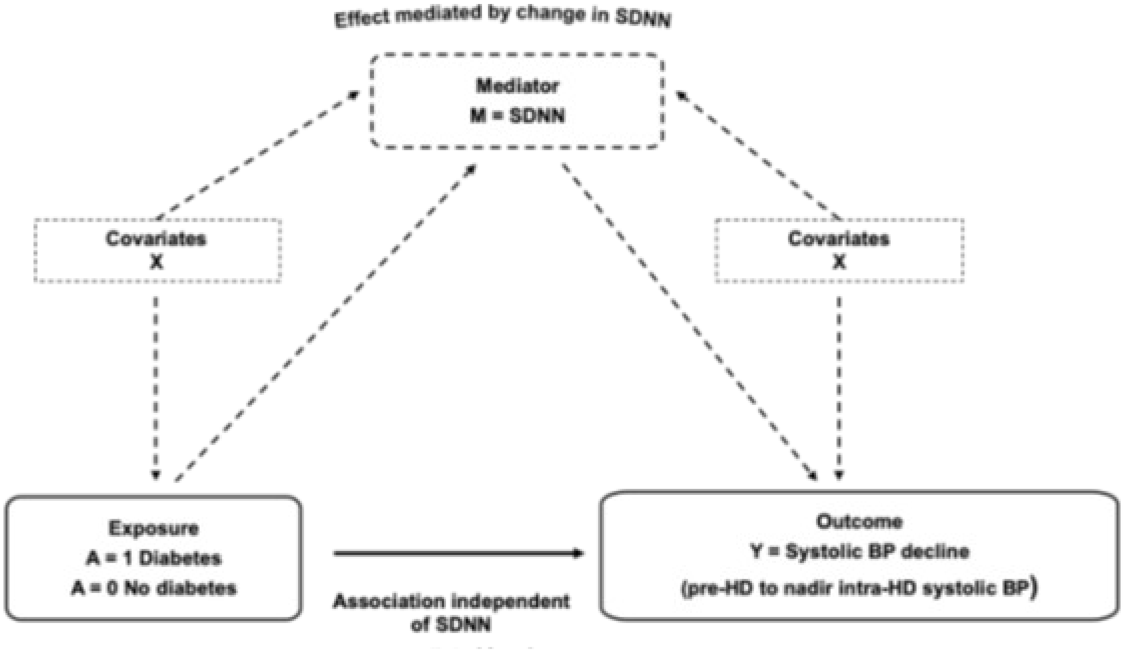
Effect mediated by change in SDNN
Mediator
M = SDNN
Covariates
X
Covariates
X
Exposure
A = 1 Diabetes
A = 0 No diabetes
Outcome
Y = Systolic BP decline
(pre-HD to nadir intra-HD systolic BP)
Association independent
of SDNN
